# Bipolar Radiofrequency and Non-Crosslinked Hyaluronic Acid Plus Calcium Hydroxyapatite in the Treatment of Stress Urinary Incontinence

**DOI:** 10.3390/ph17050622

**Published:** 2024-05-11

**Authors:** Piotr Kolczewski, Mariusz Łukaszuk, Aneta Cymbaluk-Płoska, Mateusz Kozłowski, Sylwester Ciećwież, Rafał Kuźlik, Nicola Zerbinati

**Affiliations:** 1 Department of Reconstructive Surgery and Gynecological Oncology, Pomeranian Medical University in Szczecin, Al. Powstańców Wielkopolskich 72, 70-111 Szczecin, Poland; kolczewski@gmail.com (P.K.);; 2 Novique-Aesthetic and Anti-Aging Medicine Private Clinic, 80-255 Gdańsk, Poland; 3 Department of Gynecology, Endocrinology and Gynecologic Oncology, Pomeranian Medical University in Szczecin, ul. Unii Lubelskiej 1, 71-252 Szczecin, Poland; 4 Department of Perinatology, Obstetrics and Gynecology, Pomeranian Medical University in Szczecin, ul. Siedlecka 2, 72-010 Police, Poland; 5 SaskaMed Clinic, ul. Jana Nowaka-Jezioranskiego 48, 03-994 Warsaw, Poland; 6 Department of Medicine and Surgery, University of Insubria, 21100 Varese, Italy

**Keywords:** stress urinary incontinence, radiofrequency, non-crosslinked hyaluronic acid, calcium hydroxyapatite

## Abstract

Background: Stress urinary incontinence (SUI) causes both physical and psychological problems to women and their partners. Recently, vaginal radiofrequency (RF) application, as well as the administration of non-crosslinked hyaluronic acid (NCLHA) together with calcium hydroxyapatite (CaHA), has attracted attention for SUI treatment. The current, comparative study evaluated the efficacy and safety of these technologies acting separately and in a combined treatment. Methods: Sixty women with mild to moderate SUI, aged between 46 and 76 years (mean age 63.2) were divided into three groups intended for different treatments: group I, RF vaginal treatment only, group II, NCLHA plus CaHA periurethral injection only, group III, combined treatment including a single periurethral injection of NCLHA plus CaHA followed by four vaginal applications of RF at intervals of 3–5 days. The clinical effects of the treatments were evaluated by ICIQ-LUTSqol (Polish version) and UDI-6. Results: The obtained results suggest that the symptoms of SUI and the quality of life of the patients improved significantly in each group after the therapies compared to the pre-treatment levels and were more persistent in the third HA + RF group compared to the HA or the RF group.

## 1. Introduction

Stress urinary incontinence (SUI) is an involuntary loss of urine that occurs due to an increase in intra-abdominal pressure, which can be caused by a slight or vigorous movement or physical effort, such as laughing, coughing, sneezing, and running [1]. Stress urinary incontinence (SUI) can have various underlying etiologies, including inadequate pelvic organ support, alterations in the urethral closure mechanism, or a prolapse of the anterior vaginal wall [2,3]. According to the current guidelines, the first step in the treatment of SUI, especially in situations where there is no evidence of internal urethral sphincter insufficiency, is pelvic floor muscle training (PFMT), broadly defined as urogynecological rehabilitation with lifestyle changes [4]. This intervention focuses on strengthening the pelvic floor muscles (PFM) to improve their response to an increase in intra-abdominal pressure. According to the International Urogynecology Association (IUGA0 guidelines), Kegel exercises should be used as a first-line intervention for the conservative treatment of SUI [5]. Additionally, the administration of Platelet-Rich Plasma (PRP) in combination with PFMFT has been used and exhibited better results than PMFT alone [6]. Although free of side effects and complications, their effectiveness is limited. Some female patients opt for surgical treatment involving the periurethral injection of bulking agents or sling surgery involving the insertion of a polypropylene tape under the urethra from a transobturator (TOT) or transvaginal access using special tension-free vaginal tape (TVT) sling methods [7,8,9,10]. These methods are based on the integral theory of Petros and Ulmsten, according to which the normal function of the urethra and bladder neck is provided by the vagina and the connective tissue of the vagina, as well as the striated muscles and ligaments of the small pelvis. The success rate of this treatment is high, reaching values of over 90% [11]. However, like any surgical treatment, the sling methods are associated with the risk of serious complications, such as bladder or urethral damage, erosion of the tape of the vaginal wall, bladder, or urethra, retroperitoneal hematomas, iliac vascular damage, bowel damage, urethral hypercorrection, resulting in recurrent urinary tract infections and obstructive micturition, which usually requires cutting or removing the tape [12]. Not without significance is the need for hospitalization and anesthesia, which requires the presence of an anesthesiologist, with recovery lasting 4–6 weeks. Previously regarded as the gold standard for the treatment of SUI, the utility of mid-urethral slings is now extensively contested as a result of complications and patient claims [13]. In the search for minimally invasive treatment methods, researchers are currently also considering the possible impact on SUI of energy-based biomedical technologies successfully used for many years in aesthetic medicine, such as radiofrequency (RF) or lasers as well as substances like hyaluronic acids and calcium hydroxyapatite. Another interesting method of treating SUI is extracorporeal magnetic innervation (EMI) of the pelvis floor muscles (PFM). This method is based on the indirect contraction of the PFM by an external magnetic field [14,15]. In a paper presented by Mikus et al., patients treated with EMI had a lower number of UI episodes, better QoL, and higher overall satisfaction with the treatment than patients who performed only Kegel exercises over the same time period [16]. Furthermore, the cost/benefit ratio should be taken into account; in this respect, EBD modalities generate lower costs compared to surgical interventions.

By producing an electrical field in the tissue, radiofrequency devices cause charged particles to move molecules, which produces heat [17,18,19]. The current applied and the duration of the contact between the device and the tissue directly affect the quantity of heat produced in the tissue. Depending on the type of RF device, there are variations in how the electric current travels through the tissue, between the electrodes, and back to the grounding pad. By activating heat shock proteins and starting the inflammatory cascade, radiofrequency (RF) can cause fibroblasts to generate collagen and elastin at tissue temperatures between 40 and 45 °C. The vaginal tissue may tolerate temperatures up to 47 degrees centigrade without experiencing obvious thermal injury or pain [18]. A previous study was also conducted to explore the histologic effects of radiation on tissues. It was discovered that RF causes neocollagenesis and neoelastogenesis, decreases skin laxity, and increases skin mechanical strength [18]. RF was also investigated using the Viveve monopolar applicator in the vaginal ovine model. It was discovered that three months after vaginal RF application, the increased fibroblast production measured in first month was continuing [20]. Many studies demonstrated significant results of both monopolar and bipolar RF in resolving or minimizing the complaints of women with SUI [21,22,23].

Crosslinked and non-crosslinked hyaluronic acids have also been studied in stress urinary incontinence [24,25,26], both as regenerative factors—increasing collagen and elastin levels in the urethral region—and as volumetric factors—narrowing the urethral lumen after transurethral injection. Another substance with regenerative and volumetric effects is calcium hydroxyapatite (CaHA). CaHA is substance naturally present in humans and is the main mineral component of bone and teeth. In vitro and in vivo studies demonstrated that CaHA fillers are non-toxic, non-mutagenic, non-antigenic, and non-irritating. Calcium hydroxyapatite leaves microspheres that form a scaffold for ingrowing fibroblasts and increases collagen and elastin production at the injection site. CaHA-containing products have been successfully used for many years as volumetric agents in aesthetic medicine and plastic surgery [27,28,29]. In 2019, Gonzalez et al. [30] showed a synergistic effect of non-crosslinked hyaluronic acid and calcium hydroxyapatite after injection into the vaginal wall. In this randomized study, the authors found a beneficial effect at the biochemical level—an increase in collagen and elastin concentrations—at the histological level—a thickening of the vaginal epithelium—and at the clinical level—an improvement in the symptoms of urogenital atrophy, which was assessed by the VHI (Vaginal Health Index) and the ICIQ UI SF (International Consultation on Incontinence Questionnaire Urinary Short Form), evaluating stress urinary incontinence. Similarly, Zerbinati [31] demonstrated that an injection of NCLHA plus CaHA into the anterior third of the vagina wall activated collagen and elastin production at a molecular level, restoring all the vaginal functions, such as secretion, absorption, elasticity, and lubrication, as well as improving the vaginal epithelium thickness. Histological and histochemical studies showed a significant recovery of the vaginal tissue (epithelium and stroma), and these changes appeared to be related to the bio-simulative effect of NCLHA plus CaHA [32].

In our comparative study, we present the preliminary results of three different treatments for SUI. Sixty female patients with SUI were divided into three groups. Each group was subjected to a different treatment regimen: bipolar, endovaginal RF application, NCLHA periurethral injection, and RF therapy combined with NCLHA plus CaHA injection. To address the limitations of these modalities and assuming a synergistic effect of the procedures, we hypothesized that RF therapy combined with NCLHA injection would increase the total effect of the treatment of SUI, compared to RF or NCLHA application alone. There has been no study so far presenting the effect of a combined treatment of NCLHA plus CaHA and RF on SUI.

## 2. Results

The clinical characteristics at baseline are shown in Table 1.

**Figure 1:**
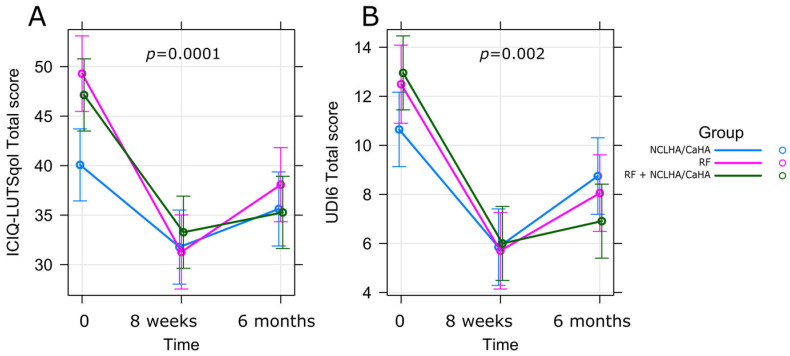
Predictor effect plots for total scores of ICIQ and UDI6. Predictor effect plots created from linear mixed-effect models for the total scores of ICIQ (**A**) and UDI6 (**B**). The *p* values correspond to time-by-group interactions.

We identified a significant group-by-time interaction for both ICIQ and UDI6 (*p* = 0.0001 and *p* = 0.002, respectively; Figure 1). To comprehensively understand the dynamics, we conducted a detailed analysis of marginal effects and contrasts within these two variables, aiming to uncover relationships and differences across distinct groups. Figure 1 illustrates predictor effect plots, providing a visual representation of trends over time in each group. Analyzing the marginal effects (Table 2), we observed a significant decrease in the ICIQ scores from baseline to the first follow-up (8 weeks) for the HA, RF, and HA + RF groups, as follows: estimate (Est.) = −8.30 (SE = 1.54), *p* < 0.001; Est. = −18.0 (SE = 1.58), *p* < 0.001; Est. = −13.9 (SE = 1.48), *p* < 0.001, respectively (Figure 1A). Similarly, the UDI6 scores exhibited a parallel trend: Est. = −4.8 (SE = 0.72), *p* < 0.001; Est. = −6.79 (SE = 0.73), *p* < 0.001; Est. −6.96 (SE = 0.69), *p* < 0.001, for the HA, RF, and HA + RF groups, respectively (Figure 1B). Conversely, from the first follow-up (8 weeks) to the second follow-up (6 months), we observed significant increases in both ICIQ and UDI6 scores, but only in the HA and RF groups (Table 2). In contrast, the changes in the RF + HA group were statistically insignificant (Est. = 2.0, SE = 1.48, *p* = 0.177 for ICIQ scores; Est. = 0.91, SE = 0.60, *p* = 0.186 for UDI6 scores).

These results indicated a significant improvement in the symptoms of SUI across all groups, as demonstrated by the reductions in the ICIQ-LUTS and UDI-6 total scores. Notably, both scores reached their lowest levels at the 8-week follow-up, showing a marked improvement shortly after treatment initiation. Although there was a slight deterioration observed at the 6-month follow-up, the observed effect of the treatments remained evident. Interestingly, the third group receiving both HA and RF treatments exhibited the most persistent improvement, suggesting a synergistic effect of these combined modalities in treating SUI.

## 3. Discussion

At various stages of life, urinary incontinence is a health concern that negatively impacts the quality of life (QOL) of women. Vaginal deliveries and estrogen deficiency during the perimenopausal phase are both risk factors for SUI. Prevalently recommended initial therapeutic interventions in that context include Kegel exercises or other exercises targeting the pelvic floor [4,33]. Nevertheless, the efficacy of physiotherapeutic exercises, including Kegel exercises, is diminished due to patients’ frequent and inconsistent failure to execute them correctly and consistently over an extended period of time (women frequently require encouragement to engage in regular Kegel exercises) [34]. Surgical procedures, on the other hand, carry a greater risk of adverse events and are more invasive, but they also have a higher likelihood of being curative [9,10,11,12].

Recently, there has been a considerable interest in the application of radiofrequency as a therapeutic approach for vaginal laxity and the genitourinary syndrome of menopause (GSM) [35,36,37,38,39,40,41]. In a similar manner, the existing literature indicates that the use of radiofrequency for the treatment of mild to moderate SUI exhibits promising results, with a negligible likelihood of adverse events [22,36,42,43,44]. In their study, Gonzalez Isaza and Velez Rizo studied the efficacy and safety of vaginal radiofrequency (RF) in the treatment of SUI [44]. It was discovered that those who met the criteria for SUI experienced a 70% clinical improvement, which was accompanied by significant changes in the ICIQ-SF scores. This was the case for 90% of the patients. According to the findings of the study, the patients exhibited a quick improvement following the completion of the treatment, and an even more significant effect was noticed during the follow-up test that was conducted four months later. According to the authors, the improvement was a result of collagen remodeling, which can take up to ninety days to complete. The urethral closing mechanism was improved as a result of this process, which may allow for an explanation for the increased reaction that was detected after four months. One can draw parallels between the data obtained and the time required for the development of new collagen. The findings of the research indicate that the method is dependable, does not cause any discomfort, is effective, and does not pose any potential adverse consequences. In order to evaluate the efficacy of monopolar and bipolar radiofrequency (RF) in the treatment of stress urinary incontinence (SUI), Abdelaziz and colleagues [22] carried out a comparative study. According to the findings of that study, the bipolar group experienced a larger improvement in the UDI-6 scores over the course of the trial compared to the monopolar group. This suggests that the treatment with bipolar RF may be more effective than the treatment with monopolar RF. Following treatment for a period of three months, the UDI-6 levels reported by the bipolar group were found to be lower than those observed in the monopolar group. Nevertheless, the UDI-6 scores increased in both groups six months after therapy, which indicates that the effectiveness of the treatment decreased over time. The values of the UDI-6 for the group with bipolar disorder were consistently lower than those for the group with monopolar disorder. The results of a safety study were comparable to those of a pilot trial that was carried out by Millheiser et al. [36], which demonstrated that radiofrequency therapy on the vaginal introitus for vaginal laxity resulted in an overall improvement of 87%, while having no adverse effects. Similarly, bipolar radiofrequency was shown to be both safe and effective in treating vaginal laxity and urogenital atrophy, as revealed by Kolczewski and colleagues during their study [41]. Bipolar RF seems to be safer than monopolar RF. In the bipolar mode, the current travels in a well-defined area, between two rings—active and passive—which are fixed to the handpiece. In contrast, in the monopolar mode, the current flows between an active electrode, which is located on the handpiece, and the grounding pad under the patient’s back and hence, it flows in a not too well defined area.

Furthermore, in our study, patients who received intravaginal bipolar RF therapy reported an improvement in stress urinary incontinence (SUI) with no side effects, as evidenced by significant improvements in the ICIQ-SF and UDI-6 scores at the 8-week follow-up. However, a slight deterioration in these improvements was observed at the 6-month follow-up, indicating a gradual return of the symptoms over time. The return of the symptoms was the slowest in the combined-treatment group, as demonstrated by the non-significant change from the 8-week to the 6-month follow-up in both ICIQ-SF and UDI-6 scores.

In the search of non-invasive methods of SUI treatment, non-crosslinked hyaluronic acid and calcium hydroxyapatite injections into the vaginal wall were also studied [30,31,32]. The authors demonstrated an improvement in SUI symptoms, as assessed by ICIQ UI SF after a single injection of NCLHA plus CaHA. Zerbinati [31] hypothesized that once the thickness of the vaginal epithelium is re-established at the level of the anterior vaginal wall, the coaptative mechanism of the urethra is restored, resulting in subjective and objective improvement in urinary incontinence. Our group receiving only the NCLHA plus CaHA single periurethral injection also showed an improvement in SUI symptoms evaluated by ICIQ-SF and UDI-6, which persisted for up to 6 months after treatment (Figure 1, Table 2).

Taking into account the growing volume of data confirming the efficacy and safety of RF therapy in SUI treatment as well as the emerging evidence concerning the effects of a SUI treatment based on the use of non-crosslinked hyaluronic acids and CaHA, we hypothesized that RF therapy combined with NCLHA plus CaHA would increase the total effect of the treatment of SUI, when compared to RF or NCLHA plus CaHA alone. Indeed, in the group receiving the combined treatment, we demonstrated the greatest improvement in SUI, as proven by the ICIQ-SF and UDI-6 scores, which was further more significant after 6 months from the treatment. So far, there has been no study presenting the effect of a combined treatment of NCLHA plus CaHA and RF on SUI.

We acknowledge the absence of blinding for both patients and evaluating doctors as a limitation of our study. Due to the nature of the treatments, implementing a double-blind design was challenging. This limitation, along with the absence of a placebo arm, is now clearly stated in the manuscript. We recognize that these factors constitute a major weakness, potentially impacting the study’s objectivity and the interpretation of the results.

We also understand the study’s low power and the reliance on subjective criteria for evaluating treatment effectiveness. The study was designed as a preliminary investigation into the potential benefits of these treatments for SUI and as such, was constrained by resource availability and the exploratory nature of the research. We, hence, agree that the study findings should be interpreted with caution and not used as a basis for clinical recommendations without further research.

We realize that some controversy surrounding RF therapy for SUI persists due to the absence of randomized controlled trials and standardized protocols. Our study, like the majority of those published, was conducted on a comparatively limited number of patients, a characteristic that diminishes its scientific validity. This is the reason why the described therapies continue to be absent from treatment recommendations. RCTs and large-scale clinical trials evaluating this technology should be conducted in the future.

## 4. Materials and Methods

The study was approved by the Research Ethics Committee under number 13/2022 and conducted according to the guidelines of the 2000 Declaration of Helsinki, updated in 2008.

**Figure 2:**
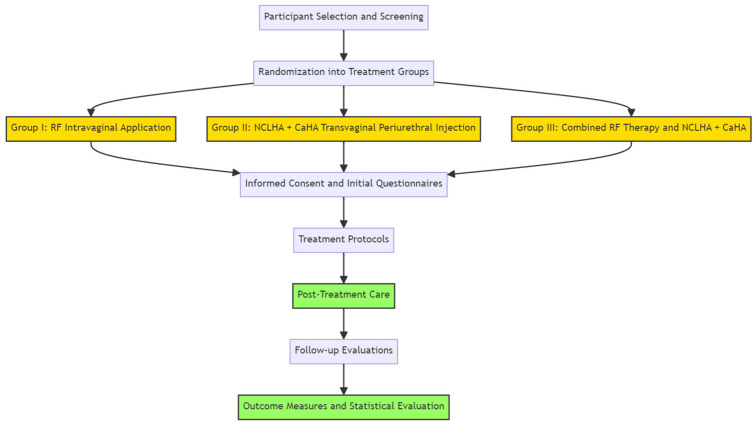
Flowchart illustrating the overall workflow of the study.

The sample included 60 females (screened and referred by a gynecologist) with mild and moderate SUI, as evaluated by the Stamey incontinence score [45]. Their ages ranged from 46 to 72 years, and their body mass index ranged from 30 to 32 kg/m^2^. Following an explanation of the treatment procedure and the nature of the study, the sample was randomly divided into 3 groups which were subjected to three different treatments, as follows (Figure 2):

- Group I—RF intravaginal application;
- Group II—NCLHA plus CaHA transvaginal periurethral injection;
- Group III—RF therapy combined with NCLHA plus CaHA injection.

The inclusion criterion was participants suffering from mild or moderate SUI due to pelvic floor weakness or hypermobility of the urethra. Participants were excluded if they had congenital urological disease, neuropathy and neurogenic bladder, detrusor hyperactivity, bladder, urethra, or vagina surgical interference, pelvic organ prolapse >2 according to the Pelvic Organ Prolapse Quantification System (POP-Q), ongoing anti-platelet treatment. Due to the use of a radiofrequency current, patients with pacemakers, metal implants, or heart rhythm disorders were excluded from the study as well.

### 4.1. Study Protocol

Following history taking and physical examination, the patients were instructed about the procedure and, after receiving and signing an informed consent, they answered the following two questionnaires: ICIQ-LUTSqol (Polish version) and UDI-6. The patients were evaluated with the same questionnaires after 8 weeks and 6 months. The treatment course was dependent on the group allocation, as described above.

### 4.2. Radiofrequency

The RF treatment was performed in groups I and III. The schedule included four treatment sessions separated by three to four days. We used the disposable gynecological applicator with a bipolar radio frequency Sectum 360 degrees (Berger & Kraft Medical Sp. z o. o., Warsaw, Poland). Each RF therapy session included vaginal RF application. In group III, after the first RF application, periurethral NCLA plus CaHA was administered as an additional procedure. The energy set up was between 5 and 10 W and was adjusted based on patient feedback (comfortable or uncomfortable).

The intravaginal application involved placing an applicator into the vaginal canal behind the hymenal ring and warming the vaginal wall over the length of approximately 4 cm over the course of 8 min. The energy setup was between 15 and 20 W and was adjusted based on patient feedback. The current was released in a pulse mode (500 ms), which means that there was no need to move the applicator back and forth in the vaginal canal. The continuous mode, which is commonly used for RF vaginal handpieces, requires that the handpiece be in constant motion to avoid burning the vaginal wall and hence can be embarrassing for both the patient and the doctor performing the procedure. To affect particularly the urethra and the periurethral region during the procedure, the tip of an applicator (two-ring part) was placed for 4 min on both sides of the urethra, exerting a slight pressure of the tip towards the anterior vaginal wall. We believe that this pressure reduces the distance between the surface of the vaginal wall and the periurethral region.

Before each RF procedure, a gel specially designed for this purpose, containing glycerin without water, was applied (Neauvia Gel).

### 4.3. Non-Crosslinked Hyaluronic Acid and Calcium Hydroxyapatite (NCLAHA plus CaHA)

The procedure was performed in groups II and III as a single transvaginal periurethral injection. In group III, for which the procedures were combined, the RF treatment was the first procedure applied.

In our study, we used a product that had been extensively examined. It is a sterile, pyrogen-free gel of NCLH, containing 18 mg/mL of pure HA, enriched with 0.01% of calcium hydroxyapatite (CaHA) plus glycine and L-proline (Neauvia Hydro Deluxe, Matex Lab SA, Geneva, Switzerland). The injection was performed transvaginally in the anterior vagina wall, laterally to the mid urethra, so as to inject the material in the periurethral region with a 27 G/37 mm disposable, blunt microcannula. The first step involved anesthetizing the patient with a cream containing 5% lidocaine and prilocaine (Emla AstraZeneca) and, after decontamination of the region, 2 mL of the product was injected on each side.

### 4.4. Post-Treatment Indications

The patients were advised that they could return to their normal activities just after the treatment. We only recommended abstaining from sexual intercourse up to 48 h after the treatment.

### 4.5. Outocome Measures and Statistic Evaluation

The ICIQ-LUTS QoL is a specialized questionnaire with 19 items that assess various areas of daily living relating to bladder dysfunction, such as leaking. The sum of these scores ranges from 19 to 76 points. A higher score suggests a lower quality of life (QoL) [46].

UDI-6 is the concise form of a condition-specific quality-of-life assessment tool [47,48]. It assesses the existence, intensity, and related urogenital symptoms of UI and classifies UI. UDI-6 comprises six components. The patients indicated the severity of their urinary distress symptoms on a scale from 0 (“no symptoms”) to 4 (“quite a bit”). The scale score (ranging from 0 to 100) was calculated by multiplying the mean scores of the answered items within each component by 25. Increased scores in the UDI-6 correspond to greater levels of impairment.

### 4.6. Statistical Analysis

Composite scores for the ICIQ and UDI6 were computed by summing the individual scores, resulting in comprehensive total scores. The baseline total scores of the HA, RF, and RF + HA groups were compared using the Kruskal–Wallis test, with post-hoc testing conducted using the Wilcoxon test and the Holm method to account for multiple comparisons. Temporal changes within and between the groups were evaluated using a mixed-effects model, implemented through the R lme4 package (version 1.1-31). The interaction between time and treatment group was examined by employing nested models and the likelihood ratio test. To aid in the interpretation of the discerned trends, predictor effect plots were generated using the R effects library (version 4.2-2). These plots served as an effective means to illustrate the impact of time and treatment group on the outcome variables. Timepoint-wise comparisons for distinct groups were conducted using the marginal effects library (version 0.15.1) in R. All statistical analyses and graphical representations were performed within the R environment, version 4.2.2 (R Core Team, 2022). The visualization of the results was accomplished using the ggplot2 library (version 3.4.0).

## 5. Conclusions

In conclusion, the patients treated with only intravaginal bipolar RF therapy reported an improvement in stress urinary incontinence (SUI), as evidenced by significant improvements in the ICIQ-SF and UDI-6 scores at the 8-week follow-up, with a slight deterioration in these improvements observed at the 6-month follow-up, indicating a gradual return of the symptoms over time.

Also, our group receiving only the NCLHA plus CaHA single periurethral injection showed an improvement in SUI symptoms, as evaluated by ICIQ-SF and UDI-6, which persisted for up to 6 months after treatment.

The group receiving a combination of RF vaginal treatment and NCLHA periurethral injection exhibited the biggest improvement in SUI, proven by the ICIQ-SF and UDI-6 scores, which was further more significant after 6 months from the treatment. This finding confirmed our assumption of a synergy between the two modalities.

## Figures and Tables

**Table 1:** Clinical characteristics at baseline.

	HA	RF	HA + RF	*p*	Post-hoc
ICIQ	40.0 ± 7.30	49.4 ± 11.0	47.1 ± 11.4	0.010	HA vs. RF, *p* = 0.009 HA vs. RF + HA, *p* = 0.063 RF vs. RF + HA, *p* = 0.601
UDI6	10.6 ± 3.5	12.5 ± 3.3	13.0 ± 3.9	0.073	HA vs. RF, *p* = 0.145 HA vs. RF + HA, *p* = 0.145 RF vs. RF + HA, *p* = 0.823

Mean ± standard deviation, *p*—Kruskal–Wallis test, post-hoc test—Wilcoxon test with Holm method to adjust the *p* values for multiple comparisons.

**Table 2:** Contrast and marginal effect analysis.

Questionnaire	Group	Contrast	Estimate	*p*
ICIQ	HA	8 weeks vs. baseline difference	−8.30 (−11.32, −5.28)	<0.001
	RF		−18.0 (−21.11, −14.91)	<0.001
	HA + RF		−13.9 (−16.77, −10.96)	<0.001
	HA	6 months vs. baseline difference	3.85 (0.81, 6.90)	0.0132
	RF		6.8 (3.75–9.85)	<0.001
	HA + RF		2.0 (−0.90, 4.90)	0.177
UDI6	HA	8 weeks vs. baseline difference	−4.8 (−6.20, −3.40)	<0.001
	RF		−6.79 (−8.23, −5.35)	<0.001
	HA + RF		−6.96 (−8.30, −5.61)	<0.001
	HA	6 months vs. baseline difference	2.9 (1.49, 4.31)	<0.001
	RF		2.35 (0.94, 3.76)	0.001
	HA + RF		0.91 (−0.44, 2.26)	0.186

Estimate—the predicted change in the dependent variable (ICIQ or UDI6) associated with each contrast, with 95% confidence interval in parentheses.

## Data Availability

The data presented in this study are available from the author P.K., upon reasonable request.
